# First isolation of *Prototheca bovis* from a bottlenose dolphin (*Tursiopstruncatus*)

**DOI:** 10.1016/j.mmcr.2025.100746

**Published:** 2025-10-24

**Authors:** Chika Shirakata, Kanon Tsurumi, Koichi Makimura, Rui Kano

**Affiliations:** a Enoshima Aquarium, 2-19-1, Katasekaigan, Fujisawa, Kanagawa, 251-0035, Japan; b Laboratory of Veterinary Physiology, Tokyo University of Agriculture and Technology, 3-5-8 Saiwai-cho, Fuchu, Tokyo, 183-8509, Japan; c Teikyo University Institute of Medical Mycology (TIMM), 359 Otsuka, Hachioji, Tokyo, 192-0395, Japan

**Keywords:** Algaecid effect, Anti-fungal drugs, Bottlenose dolphin, Susceptibility, Prototheca bovis

## Abstract

To our knowledge, this is the first report of *Prototheca* sp. isolated from the digestive tract of a dolphin. A captive-born female bottlenose dolphin (*Tursiops truncatus*) weighing 165 kg that was housed at Enoshima Aquarium presented with a slightly elevated body temperature and candida-like yeasts in stomach fluid and feces. Gastroscopy revealed cobblestone-like thickening of the mucosa with a few ulcers in the forestomach. *Prototheca bovis* was isolated from the biopsy specimen of the lesion.

## 1 Introduction

The genus *Prototheca* comprises achlorophyllic algae that are ubiquitous in the environment and in the intestines of animals. However, *Prototheca* spp. have lost their ability to photosynthesize and have adopted a sometimes parasitic lifestyle. Certain species are capable of causing protothecosis in humans and animals, with *P. wickerhamii* and *P. bovis* most commonly associated with disease [[1], [2], [3]]. However, little is known about the pathogenicity of *Prototheca* in marine animals. To our knowledge, this is the first report of *Prototheca* sp. isolated from the digestive tract of a dolphin.

## 2 Case presentation

**Fig. 1:**
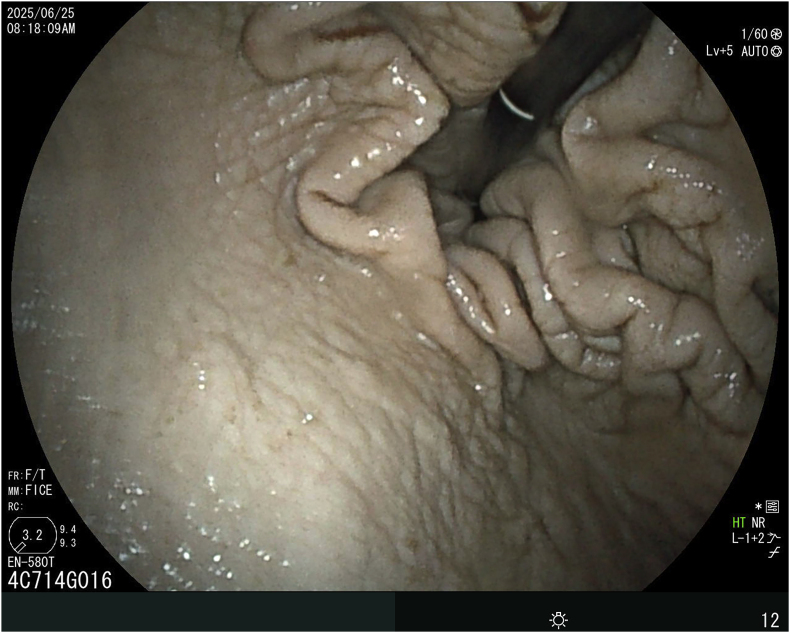
Forestomach gastroscopy image of a 13-year-old bottlenose dolphin from which *Prototheca bovis* was cultured from gastric fluid. Cobblestone-like mucosal thickening and ulcers are visible.

A captive-born 13 years old female bottlenose dolphin (*Tursiops truncatus*) weighing 165 kg that was housed at Enoshima Aquarium presented with a slightly elevated body temperature (37.3 °C) and *Candida*-like yeasts in stomach fluid and feces on day 1 (Table 1). Oral itraconazole (ITCZ) treatment was initiated at a dosage of 2.5 mg/kg twice daily (BID). As the low-grade fever persisted after 21 days of itraconazole treatment, an endoscopic examination was performed. Gastroscopy revealed cobblestone-like thickening of the mucosa with a few ulcers in the forestomach, but no lesions were observed in the main stomach on day 21 (Fig. 1). After the isolate was identified as a *P. bovis*, the oral ITCZ treatment was stopped because *Candida* and *Prototheca* were considered saprophytes. Treatment with antibiotics was started. Since the low-grade fever persisted for 4 weeks (on day 99), a second endoscopic examination and culture were conducted. Mucositis of forestomach was observed, but *P. bovis* was not isolated again. *P. bovis* and *C. albicans* were isolated from the lesion and fos-ravuconazole (F-RVCZ) was started (2mg/kg once daily, orally) to treat *Protothea* infection. After 26 days of treatment with F-RVCZ, the body temperature was within the normal range and the condition was improved.

**Table 1:** History of the case.

Day	Topic	Isolation from forestomach	Treatment
1	Fever (37.3 °C) and loss of appetite		ITCZ (2.5 mg/kg twice daily, orally)
21	Normal body temperature (36.6 °C) A few ulcers in the forestomach	*C. albicans* and *P. bovis*	Candida and Prototheca were considered saprophytes. ITCZ was stopped. Oral antibiotic treatments were started.
25	Fever (37.5 °C) and feeling down		Continue with antibiotics.
99	Fever (37.9 °C) and feeling down Ulcers in the forestomach		F-RVCZ (2mg/kg once daily, orally) was started.
106		*C. albicans* and *P. bovis*	Continue with F-RVCZ.
113		*C. albicans*	Continue with F-RVCZ.
120	Normal body temperature (37.0 °C) Condition improvement	*C. albicans*	Continue with F-RVCZ.
124			F-RVCZ treatment was stopped.

ITCZ: itraconazole, F-RVCZ: fos-ravuconazole.

**Fig. 2:**
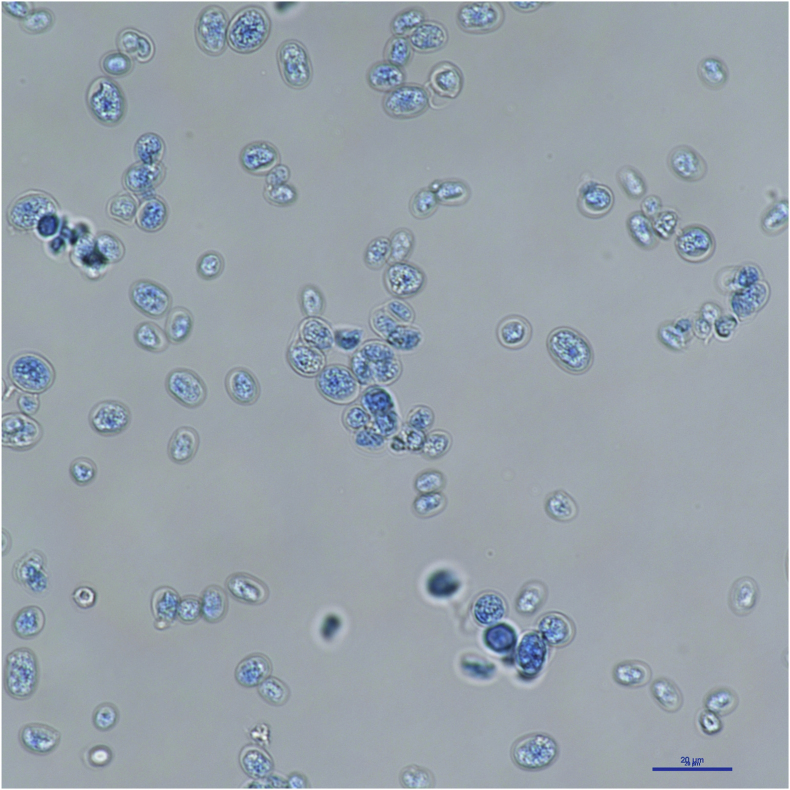
Microscopic examination of the isolate showing numerous spheroid sporangia measuring 7–15 μm in diameter, as well as elongated and slightly lunate forms, with sporangiospores measuring 3–7 μm in diameter.

A biopsy specimen from the surface of the forestomach was inoculated onto CHROMagar™ Candida (Kanto Chemical Co., Inc., Tokyo, Japan) and incubated at 32 °C. After 4 days of incubation, numerous wet, white-to-purple colonies developed on the medium surface. Microscopic examination of the isolates revealed numerous spheroidal sporangia measuring 7–15 μm in diameter, as well as elongated and slightly lunate forms, with sporangiospores measuring 3–7 μm in diameter (Fig. 2). Based on these morphological characteristics, the isolates were presumptively identified as *Prototheca* spp [4]. Molecular identification of the strain was determined by 18S rDNA sequence analysis. A PCR product of approximately 520 bp was obtained and sequenced [5]. Comparative nucleotide sequence analysis revealed 100 % identity with *Prototheca bovis* (strain SAG 2021 18S ribosomal RNA gene, complete sequence; GenBank accession no. MF163508). The isolate was therefore identified as a *P. bovis*. Minimum inhibitory concentrations (MICs) for antialgae agents were determined based on the Clinical and Laboratory Standards Institute (CLSI) M27-A3 guidelines [6,7]. The MIC values for the isolate were <0.03 μg/ml for amphotericin B, 1 μg/ml for ITCZ, 1 μg/ml for voriconazole, <0.03 μg/ml for ravuconazole, 0.25 μg/ml for posaconazole, and 0.5 μg/ml for micafungin (Table 2).

**Table 2:** Susceptibility of P. bovis and C. albicans to antifungal drugs and phytochemicals.

Species (Days^a^)	MICs (mg/l)						
	AMB	FLCZ	ITCZ	RVCZ	PSCZ	VRZ	MYC
*P. bovis (21)*	0.06	>64	1	0.06	0.25	1	4
*P. bovis (106)*	0.125		16	0.125	2	8	
*C. albicans (120)*	0.06	8	<0.03	<0.03	<0.03	<0.03	<0.03

MICs: minimum inhibitory concentrations.

AMB: amphotericin B, FLCZ: fluconazole, ITCZ: itraconazole, RVCZ: ravuconazole, VRZ: voriconazole, MYC: micafungin.

a Isolation days.

## 3 Discussion

The sequence of the 18S ribosomal rDNA of the isolates was 100 % identical to that of the type strain of *P. bovis* and isolates derived from bovine mastitis, suggesting that they were the same as *P. bovis* isolated from the cows. Bovine protothecal mastitis, defined as an inflammation of the mammary gland, is the most prevalent and devastating disease in dairy cattle worldwide [1]. *P. bovis* is most associated with bovine mastitis, a disease that causes a reduction in milk production and secretion of thin watery milk containing white flakes. *P. bovis* (former species was *P. zopfii*) sometimes causes cutaneous or disseminate protothecosis in humans and small animals [[1], [2], [8]]. Among Japanese cases, cutaneous cases were most frequently reported and showed the best prognosis, while disseminated cases were rare and had the worst prognosis [2].

This is the first report of *P. bovis* isolated from dolphins, but it was not clear whether this *Prototheca* had infected the dolphins. The oral ITCZ was administered based on a provisional diagnosis of candidiasis in the dolphin. However, the MIC (1 μg/ml) of ITCZ for the *P. bovis* suggests the treatment was likely ineffective (Table 2). However, following treatment with RVCZ, *P. bovis* was no longer isolated, and the dolphin's condition improved, suggesting protothecosis for the dolphin. RVCZ is an available human azole drug against human onycomycosis in Japan since 2018 and exhibits excellent *in vitro* anti-algae activity against *Prototheca* species [7]. RVCZ was more potent than the other azoles against *Prototheca* species and has considerable potential for use as a therapeutic agent for human and animal protothecosis [7].

Notably, *P. bovis* was isolated from any of the other dolphins from the culture examination of gastroscopy samples. Therefore, these findings suggest that *P. bovis* was present in the seawater but was not highly pathogenic for dolphins. We speculated that the opportunistic infection resulted from candidiasis treatment and compromised immunity.

Protothecosis is considered one of the zoonosis which means animal to human transmission or environment to human and animals [9]. Human health risk from consumption of processed milk is low, as pasteurization is usually effective against *Prototheca* [9]. Wang et al., investigated case reports on disseminated protothecosis in humans concluded that the skin was not only the primary site of entry but also the organ most frequently affected by the pathogen [10]. Therefore, there is a possibility of *Prototheca* infection of this dolphin case through skin wounds or oral uptake of the seawater. Because persistence and proliferation of *Prototheca* spp. in aquatic environments is well documented for sea water [9]. However, since the ecology of *Prototheca* spp. in marine environments and epidemiology in marine mammals remain poorly understood, further investigation into protothecosis in marine mammals may be warranted within a One Health approach.

## CRediT authorship contribution statement

**Chika Shirakata:** Formal analysis. **Kanon Tsurumi:** Investigation. **Koichi Makimura:** Writing – review & editing, Investigation. **Rui Kano:** Writing – original draft, Investigation, Data curation.

## Ethical statement of “first isolation of *Prototheca bovis* from a bottlenose dolphin (*Tursiops truncatus*)”

This study was conducted according to the principles of the Declaration of Helsinki.

## Conflict interest

No other author has reported a potential conflict of interest relevant to this case report.

## References

[1] Todd J.R., Matsumoto T., Ueno R., Murugaiyan J., Britten A., King J.W. (2018). Medical phycology 2017. Med. Mycol..

[2] Kano R. (2020). Emergence of fungal-like organisms: *prototheca*. Mycopathologia.

[3] Jagielski T., Bakula Z., Gawor J., Maciszewski K., Wolf-Henning, Kusber W. (2019). The genus *Prototheca* (Trebouxiophyceae, Chlorophyta) revisited: implications from molecular taxonomic studies. Algal Res..

[4] Kano R., Satoh K., Yaguchi T., Masuda M., Makimura K., de Hoog G.S. (2022). Phenotypic characteristics of *Prototheca* species occurring in humans and animals. Med. Mycol. J.

[5] Sobukawa H., Yamaguchi S., Kano R., Ito T., Suzuki K., Onozaki M. (2012). Short communication: molecular typing of *Prototheca zopfii* from bovine mastitis in Japan. J. Dairy Sci..

[6] Clinical Laboratory Standards Institute. CLSI document M27-A3 (2008).

[7] Miura A., Kano R., Ito T., Suzuki K., Kamata H. (2020). *In vitro* algaecid effect of itraconazole and ravuconazole on *Prototheca* species. Med. Mycol..

[8] Jagielski T., Proskurnicka A., Iskra M., Wronka S., Bakuła Z., Danesi P. (2025). Protothecosis in dogs: a narrative review. J. Vet. Intern. Med..

[9] Libisch B., Picot C., Ceballos-Garzon A., Moravkova M., Klimesová M., Telkes G., Chuang S.T., Le Pape P. (2022). *Prototheca* infections and ecology from a one health perspective. Microorganisms.

[10] Wang X., Ran Y., Jia S., Ahmed S., Long X., Jiang Y. (2022). Human disseminated protothecosis: the skin is the "window". Front. Immunol..

